# A massive compact quiescent galaxy at *z* = 2 with a complete Einstein ring in JWST imaging

**DOI:** 10.1038/s41550-023-02103-9

**Published:** 2023-10-19

**Authors:** Pieter van Dokkum, Gabriel Brammer, Bingjie Wang, Joel Leja, Charlie Conroy

**Affiliations:** 1 Department of Astronomy, Yale University, New Haven, CT USA; 2 Cosmic Dawn Center (DAWN), Copenhagen, Denmark; 3 Niels Bohr Institute, University of Copenhagen, Copenhagen, Denmark; 4 Department of Astronomy & Astrophysics, The Pennsylvania State University, University Park, PA USA; 5 Institute for Computational & Data Sciences, The Pennsylvania State University, University Park, PA USA; 6 Harvard-Smithsonian Center for Astrophysics, Cambridge, MA USA

**Keywords:** Galaxies and clusters, Stars

## Abstract

One of the surprising results from the Hubble Space Telescope was the discovery that many of the most massive galaxies at redshift *z* ≈ 2 are very compact, having a half-light radius of only 1−2 kpc. The interpretation is that massive galaxies formed inside out, with their cores largely in place by *z* ≈ 2 and approximately half of their present-day mass added later through minor mergers. Here we present a compact, massive, quiescent galaxy at a photometric redshift of ${z}_{{{{\rm{phot}}}}}=1.9{4}_{-0.17}^{+0.13}$ with a complete Einstein ring. The ring was found in the James Webb Space Telescope COSMOS-Web survey and is produced by a background galaxy at ${z}_{{{{\rm{phot}}}}}=2.9{8}_{-0.47}^{+0.42}$. Its 1.54*″* diameter provides a direct measurement of the mass of the ‘pristine’ core of a massive galaxy, observed before the mixing and dilution of its stellar population during the 10 Gyr of galaxy evolution between *z* = 2 and *z* = 0. We find a mass for the lens ${M}_{{{{\rm{lens}}}}}=6.{5}_{-1.5}^{+3.7}\times 1{0}^{11}$ *M*_⊙_ within a radius of 6.6 kpc. The stellar mass within the same radius is ${M}_{{{{\rm{stars}}}}}=1.{1}_{-0.3}^{+0.2}\times 1{0}^{11}$ *M*_⊙_ for a Chabrier initial mass function and the fiducial dark matter mass is ${M}_{{{{\rm{dm}}}}}=2.{6}_{-0.7}^{+1.6}\times 1{0}^{11}$ *M*_⊙_. Additional mass appears to be needed to explain the lensing results, either in the form of a higher-than-expected dark matter density or a bottom-heavy initial mass function.

## Abstract (web summary)

A rare perfect alignment between two galaxies in the young Universe has been captured by the James Webb Space Telescope. The further (*z* ≈ 3) galaxy is curved into an Einstein ring due to the bending of space around the nearer (*z* ≈ 2) galaxy, which is massive and compact—representative of the pristine core of a present-day galaxy.

## Main

The galaxy and its ring were identified in James Webb Space Telescope (JWST) NIRCam observations in the context of the COSMOS-Web project^1^, a public wide-area survey using the F115W, F150W, F277W and F444W filters. A visual inspection of a mosaic generated from the F115W, F277W and F444W data available as of 15 April 2023, covering a total area of 0.35 deg^2^, readily revealed the object (Methods). The NIRCam images containing the galaxy were resampled^2^ to a common 0.025*″* per pixel grid for analysis.

**Fig. 1:**
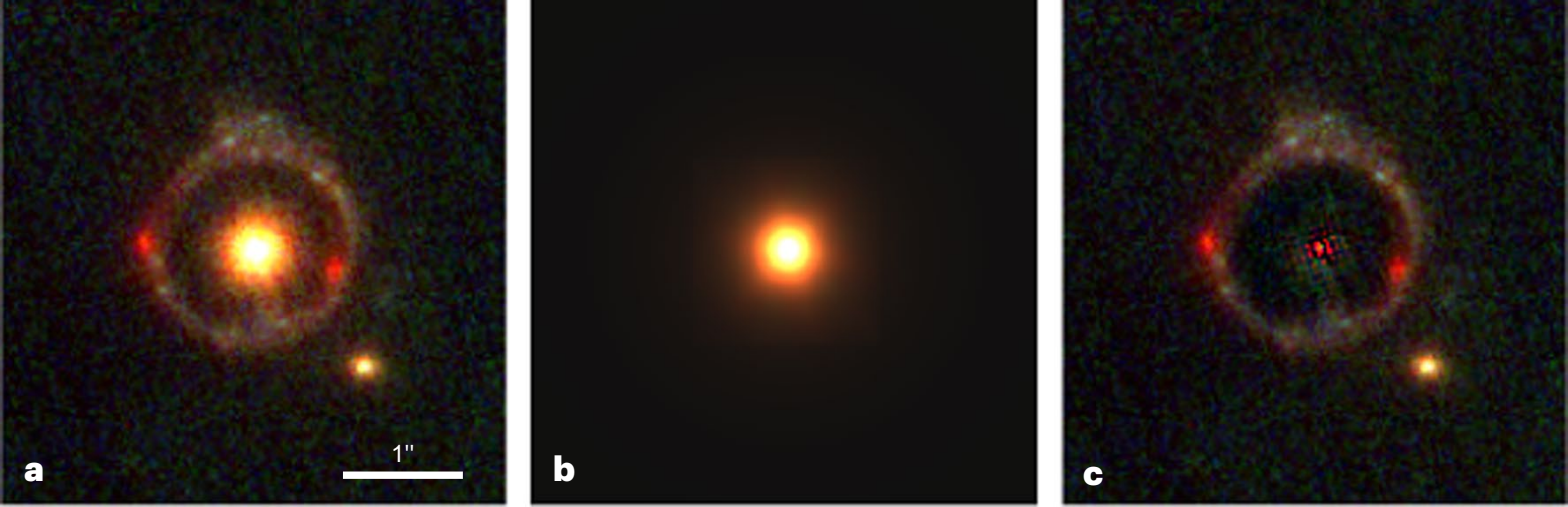
A complete Einstein ring identified in JWST images. **a**, Colour image of JWST-ER1, created from the NIRCam F115W, F150W and F277W data. **b**, Model of the galaxy, with an effective radius of *r*_e_ = 1.9 kpc. **c**, Residual of the fit. Each panel spans 4.1*″* × 4.1*″*. The coordinates of the lens are RA = 10 h 00 min 24.11 s, ${{{\rm{dec.}}}}={1}^{\circ }\,5{3}^{{\prime} }\,34.{9}^{{\prime}{\prime} }$ (J2000).

The object, dubbed JWST-ER1, is shown in Fig. 1a. It consists of a compact early-type galaxy (JWST-ER1g) and a complete Einstein ring (JWST-ER1r) with two conspicuous red concentrations. The lensed galaxy probably has a red centre and a blue disk, with parts of the disk producing the ring. The diameter of the centre of the ring is 1.54*″* ± 0.02*″*. JWST-ER1 joins a large number of known Einstein rings^3,4^, although most are not complete. Like other strong lensing configurations, Einstein rings can be used to reconstruct high-resolution images of lensed background galaxies, using ray-tracing techniques^5^. However, the unique value of Einstein rings is what they tell us about the lenses themselves: given the redshifts of the lens and source, they provide a model-independent measurement of the enclosed mass within the radius of the ring^6^.

**Fig. 2:**
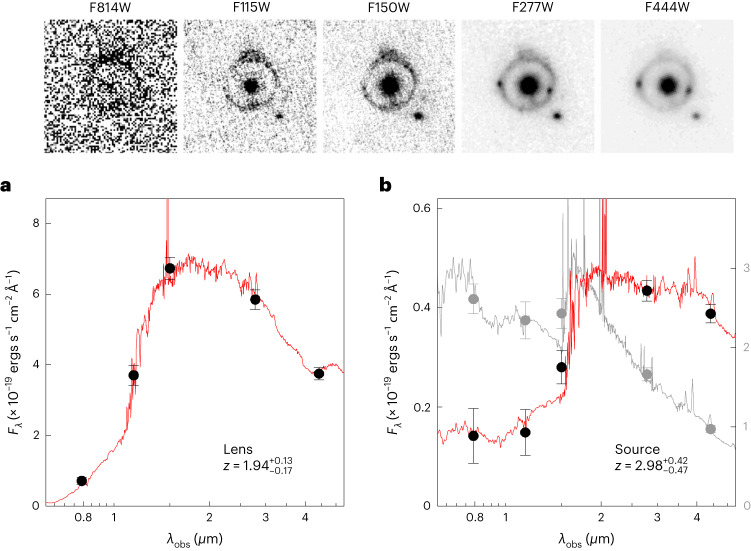
Photometry of the lens and source. Top panels: images in the HST/ACS F814W band and the JWST/NIRCam F115W, F150W, F277W and F444W bands. They are shown at a common *F*_*λ*_ scale. **a**, SED of the lens galaxy, determined from forced GALFIT fits. The galaxy is well fit by a quiescent stellar population at $z=1.9{4}_{-0.17}^{+0.13}$ and a total stellar mass of $1.{1}_{-0.4}^{+0.3}\times 1{0}^{11}$ *M*_⊙_ (for a Chabrier IMF). **b**, SED of the lens galaxy, with the summed flux of the two red knots shown in black circles and the blue ring in grey circles. The red knots provide a reasonably well-constrained redshift of ${z}_{{{{\rm{phot}}}}}=2.9{7}_{-0.37}^{+0.44}$. Data are presented as measurements ± s.d.

We obtained five-band photometry of the lens by fitting it with a Sèrsic model^7^, masking the ring and keeping the structural parameters fixed in all bands. The effective radius of the galaxy *r*_e_ = 0.22*″* ± 0.02*″* and its Sèrsic index *n* = 5.0 ± 0.6. The total magnitudes of the galaxy are given in Table 1 and the spectral energy distribution (SED) is shown in Fig. 2a. There is a pronounced break between the F814W and F115W bands, leading to a well-constrained photometric redshift of $z=1.9{4}_{-0.17}^{+0.13}$ for the lens (Methods). The photometric redshift exceeds the spectroscopic redshift of the most distant known lens, a *z* = 1.62 galaxy in a cluster^8^. The source redshift is less well constrained. We split the source into two photometric masks, one containing the blue ring and one covering both of the red knots. The blue ring shows no strong features and has a redshift of ${z}_{{{{\rm{phot}}}}}=2.8{9}_{-0.98}^{+0.27}$. The SED of the red knots has a clear break between F150W and F277W, and a better-constrained redshift of ${z}_{{{{\rm{phot}}}}}=2.9{8}_{-0.47}^{+0.42}$ (Fig. 2b).

**Table 1:** Structural parameters of the lens

Filter	*r*_e_ (pixels)	*n*	*b*/*a*	PA
F115W	7.9 ± 0.7	4.1 ± 0.3	0.94	77
F150W	9.9 ± 0.5	4.9 ± 0.2	0.96	−15
F277W	8.8 ± 0.4	5.3 ± 0.2	0.98	−16
F444W	8.6 ± 0.4	5.6 ± 0.2	0.99	−23

*n* is the Sersic index. PA is the position angle.

The lensing galaxy appears to be a textbook example of the class of massive quiescent galaxies at *z* ≈ 2. Its rest-frame colours, U−V ≈ 2.10 and V−J ≈ 1.3, place it comfortably in the quiescent region of the *z* ≈ 2 UVJ diagram^9^. The best-fit stellar population parameters from the Prospector^10^ fit imply an age of $1.{9}_{-0.6}^{+0.3}$ Gyr and a low star formation rate of ${4}_{-3}^{+19}$ *M*_⊙_ yr^−1^. The Prospector total stellar mass of JWST-ER1g is $1.{3}_{-0.4}^{+0.3}\times 1{0}^{11}$ *M*_⊙_ for a Chabrier^11^ initial mass function (IMF), and its observed effective radius corresponds to *r*_e_ = 1.9 ± 0.2 kpc. This makes the galaxy quite compact, just like other quiescent galaxies at these redshifts^12–15^, and it falls on the canonical size–mass relation of quiescent galaxies^16^. The galaxy is almost perfectly round and there are no obvious star-forming regions, tidal tails or other irregularities in the residuals from the GALFIT fit.

We now turn to the mass of JWST-ER1g as inferred from the radius of the Einstein ring. The photometric redshifts of the lens and source, combined with the radius of the Einstein ring, give a total mass of ${M}_{{{{\rm{lens}}}}}={6.5}_{-1.5}^{+3.7}\times {10}^{11}$ *M*_⊙_ within *r* = 6.6 kpc (Methods). The stellar mass within the Einstein radius is 0.79 times the total mass as determined by GALFIT and Prospector, that is, ${M}_{{{{\rm{stars}}}}}=\left({1.1}_{-0.3}^{+0.2}\times {10}^{11}\right)$ *M*_⊙_ for a Chabrier IMF. There is a large difference between the lens mass and the Chabrier stellar mass of JWST-ER1g, with the lens mass a factor of $5.{9}_{-1.6}^{+4.1}$ higher than the stellar mass. This is the central result of our study (besides the report of the discovery of JWST-ER1), and in the following we discuss several possible contributors to the lensing mass.

It is unlikely that a significant fraction of the lensing mass is in the form of gas. Observations of lensed quiescent galaxies^17^, as well as simulations^18,19^, have consistently found low gas masses (<10^10^ *M*_⊙_) for massive quiescent galaxies at these redshifts. Furthermore, a total gas mass of 3 × 10^11^ *M*_⊙_ within 6.6 kpc corresponds to such a high projected gas density that a high star formation rate is inevitable. The average projected surface density would be ~2,200 *M*_⊙_ pc^−2^, and according to the Kennicutt–Schmidt relation^20^ the corresponding star-formation-rate surface density is *Σ* ≈ 14 *M*_⊙_ yr^−1^ kpc^−2^. The total star formation rate within the ring would be ~2,000 *M*_⊙_ yr^−1^, which is three orders of magnitude higher than derived from the Prospector fits and 30 times higher than an upper limit derived from Spitzer/MIPS 24 μm data (Methods). This is a rough estimate, with the actual star formation rate depending on the distribution and temperature of the gas, but the point is that JWST-ER1g would not be quiescent but rather a strong starburst galaxy.

**Fig. 3:**
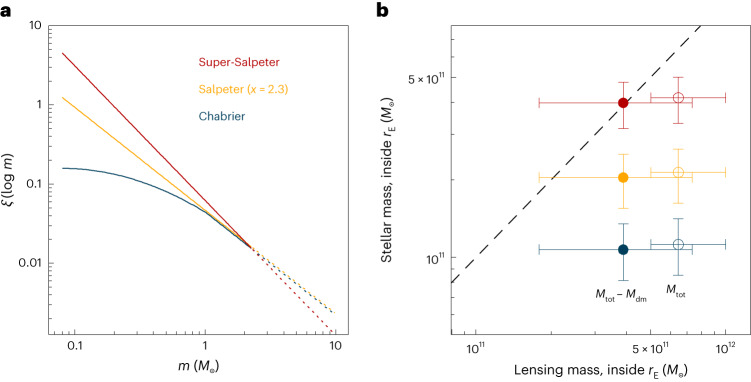
Comparison of stellar mass to lensing mass for different IMFs. **a**, The three IMFs that are considered in this study: a Chabrier IMF^11^, a Salpeter IMF^27^ and an IMF that is steeper than Salpeter with a slope of −2.7. Broken lines are for stars above the turn-off mass. **b**, Comparison of the stellar mass to the lensing mass (open symbols) and the lensing mass minus the fiducial amount of dark matter (solid symbols), for the three IMFs. The dashed line indicates a one-to-one relation. Data points are measurements ± s.d. *r*_E_, Einstein radius; *x*_i_, number of stars; *m*, mass of stars; *M*_tot_, total mass.

There is of course dark matter within the Einstein ring, and with standard assumptions this explains about half of the difference between the lensing mass and the stellar mass. Assuming a Navarro–Frenk–White (NFW) profile^21^ and the stellar mass–halo mass relation^22^ for *z* = 2, the dark matter mass within the Einstein radius is ${M}_{{{{\rm{dm}}}}}=2.{6}_{-0.7}^{+1.6}\times 1{0}^{11}$ *M*_⊙_ (Methods). As shown in Fig. 3, this leaves $2.{8}_{-2.1}^{+3.4}\times 1{0}^{11}$ *M*_⊙_ unaccounted for. An explanation for this mild discrepancy is that the dark matter density within the Einstein radius is a factor of ~2 higher than expected from scaling relations. The ‘extra’ dark matter can come in two forms. First, the total halo mass could be higher than what is indicated by the canonical stellar mass–halo mass relation. A second option is that baryonic processes have led to a dark matter profile that deviates from the NFW form. The final profile can be steeper or shallower in the central regions, depending on the balance between cooling and feedback^23–25^.

Looking closer, both options are somewhat unlikely in the specific case of JWST-ER1g. As detailed in the Methods section, the total halo mass would have to be very high, close to *M*_halo_ ≈ 10^14^ *M*_⊙_, and only a few halos of that mass are expected to exist in the surveyed volume. Turning to baryonic processes, they tend to alter the dark matter profile on the spatial scales where the baryons are located: specifically, significantly steeper profiles are expected in regions where the stellar mass dominates^24^, that is, at radii of at most *r*_e_. The dark matter mass within 1.9 kpc is only 3 × 10^10^ *M*_⊙_ for a 10^13^ *M*_⊙_ halo with a NFW profile, and even if this were enhanced by a factor of 2–3, it would not be enough to account for the missing mass within the Einstein radius.

An intriguing alternative is that the missing mass is in the form of low mass stars, and that the stellar IMF needs to be adjusted: stars with masses *M* ≈ 0.5 *M*_⊙_ and below dominate the total mass but contribute less than 5% to the light^26^. Rather than simply scaling the mass, we refit the photometry in Prospector with two bottom-heavy IMFs: the Salpeter form^27^, with a slope of −2.3 and no turnover, and a ‘super-Salpeter’ IMF with a slope of −2.7. These IMFs are illustrated in Fig. 3a. We note that these parameterizations are not unique, as the low mass slope is degenerate with the low mass cut-off. Furthermore, top-heavy IMFs can lead to high *M*/*L* ratios too if the mass is dominated by stellar remnants, although even for very flat IMFs this only occurs at ages greater than 3 × 10^9^ Gyr (ref. ^28^). With these caveats in mind, we find that the stellar mass within the Einstein radius is $2.{0}_{-0.5}^{+0.5}\times 1{0}^{11}$ *M*_⊙_ for a Salpeter IMF and $4.{0}_{-0.8}^{+0.6}\times 1{0}^{11}$ *M*_⊙_ for the super-Salpeter IMF. As shown in Fig. 3b a model that combines a super-Salpeter IMF with a standard dark matter halo matches the lensing mass exactly, with a Salpeter IMF also providing a good fit.

The probable descendants of compact quiescent galaxies at *z* ≈ 2 are massive early-type galaxies^29–32^, and the central regions of these galaxies may indeed have IMFs that are more bottom heavy than the Chabrier IMF. The evidence largely comes from gravity-sensitive absorption lines^33^, kinematics^34^ and gravitational lensing^35^. Outside the central regions there appears to be a gradual transition to a Chabrier IMF^36–39^, as expected if a significant fraction of the mass in the outskirts was accreted through minor mergers. Quantitatively, the excess stellar mass compared to a Chabrier IMF reaches a factor of ~3 in the centres of massive galaxies, with a power-law slope of −2.7 found for the galaxy NGC 1407 from a detailed non-parametric analysis^40^. Super-Salpeter slopes of −2.7 have also been proposed on theoretical grounds^41^. We infer that a steep IMF for JWST-ER1 would be consistent with estimates in the central regions of early-type galaxies, particularly when mixing and dilution due to mergers and projection effects are taken into account^42^.

While this consistency is encouraging, IMF measurements are difficult and often indirect, and the question of IMF variation in the central regions of elliptical galaxies is still debated^39,43^. Furthermore, and of direct relevance to JWST-ER1g, bottom-heavy IMFs are in some tension with comparisons of dynamical masses to stellar masses of *z* ≈ 2 galaxies, which tend to prefer bottom-light IMFs such as the Chabrier form^44,45^. On the other hand, our results are qualitatively consistent with the most similar system to JWST-ER1, which is a *z* = 1.525 lens and partial Einstein ring that is best fit with a Salpeter IMF^8^.

The combination of lensing with kinematics can break some of the degeneracies between the dark matter profile and the stellar mass, as has been demonstrated at lower redshifts^46^. This should work particularly well for JWST-ER1 as the effective radius of the galaxy is a factor of 3.5 smaller than the Einstein radius. Future NIRSpec observations of JWST-ER1 could provide the velocity dispersion of the galaxy, and pin down the redshifts of the lens and source.

## Methods

### Discovery

JWST-ER1 is located in the COSMOS-Web JWST data^1^, as described in the main text. We reduced and aligned the NIRCam images with a software pipeline that was previously developed for the Hubble Space Telescope (HST) imaging and was modified for the JWST instruments^47^. Existing HST/ACS F814W imaging from the original COSMOS project^48^ and datasets at other wavelengths were processed in the same way, so that all space-based images are aligned to a common astrometric frame. The galaxy was found from a visual inspection of a mosaic that was generated from the F115W, F277W and F444W data.

It is not the first Einstein ring that was found in the COSMOS field; there are at least two others, along with several more candidates^49^. This raises the question of why JWST-ER1 had not been noticed before. The main reason is that both the source and the lens are faint in the optical, and that existing HST data in the near-infrared—while showing the lens—are not deep enough to show the source. In Supplementary Fig. 1 the pre-JWST high-resolution data are shown: the HST/ACS F814W from the original COSMOS programme^48^ and a short-exposure HST/WFC3 F160W image from 3D-DASH, a wide-field survey with the drift-and-shift (DASH) technique^50^. With the benefit of hindsight, the characteristics of an Einstein ring can be glimpsed: a compact red galaxy near the centre of a blue ring.

### Is it a lens?

We consider the possibility that the system is not a gravitational lens but a ring galaxy, such as Hoag’s object^51^. Star-forming rings can be created in head-on collisions^52^ and there is a small galaxy to the southwest of the ring that could be the perturber. The most obvious argument in favour of the lensing interpretation is that the photometric redshift of the ring is higher than that of the central galaxy (see main text). However, the redshift of the ring is uncertain, and it might be possible to fit both the lens and the ring with a model at *z* ≈ 2.1.

Here we highlight the morphology of the ring. In Supplementary Fig. 2 we show an enlarged, high-contrast colour image generated from the F150W and F444W data, after subtracting the best-fitting model for the central galaxy. There are several symmetries in the image: as well as the two bright red knots it appears that two blue knots are also multiple imaged. The most compelling argument for lensing is the morphology of the red knots (presumably the bulge of the lensed galaxy): they are stretched into mirrored arcs on each side of the galaxy, something that cannot be explained in collisional ring scenarios.

### Structural parameters

We fit the lens galaxy with the GALFIT code^53^ to determine its structure and in preparation for measuring its photometry. We use cutouts of 4.1*″* × 4.1*″* with 0.025*″* per pixel sampling in the NIRCam bands and 0.05*″* per pixel sampling in the ACS F814W band. The presence of the ring makes it difficult to measure the size, Sèrsic index and background level simultaneously. We therefore first measure the background level in each band from the outer edge of the cutout, iteratively rejecting outlying pixels, and subtract this value. Next a mask is created for the ring, by selecting pixels in the ring area above a flux threshold and then expanding the mask using a 5 × 5 pixel kernel.

The fit is performed on the F115W, F150W, F277W and F444W images (the signal-to-noise ratio in the F814W image is too low for a stable fit). Free parameters are the position, Sèrsic index, effective radius, total magnitude, axis ratio and position angle. We use the WebbPSF tool (https://www.stsci.edu/jwst/science-planning/proposal-planning-toolbox/psf-simulation-tool) to create point spread functions (PSFs) for each filter and position. We verified that a well-exposed nearby star does not lead to qualitatively different results.

The structural parameters are listed in Table 1. The parameters in the four bands are in good agreement, despite the factor of 4 range in wavelength and resolution going from F115W to F444W. The average effective radius *r*_e_ = 8.8 ± 0.8 pixels, or 0.22*″* ± 0.02*″*, where the root mean square of the four individual measurements is taken as the uncertainty. The Sèrsic index *n* = 5.0 ± 0.6. The axis ratio is very close to 1 and there is no consistent position angle between the bands; in what follows we therefore assume that the axis ratio *b*/*a* = 1.0.

### Photometry

Total magnitudes of the lens are determined by fitting the five bands (now including ACS F814W) with GALFIT, holding all parameters except the total magnitude fixed to the average values determined above. This constrained (or forced) fit ensures that the relative fluxes between the bands are measured in a self-consistent way, and not compromised by PSF or aperture effects. The results are listed in Supplementary Table 1, with 0.05 mag systematic error added in quadrature to the random errors. For the comparison of the lensing mass to the stellar mass it is not the total flux but the projected flux within the Einstein radius that matters. Using a model profile that is not convolved with the PSF we determine that 79% of the total flux is within the Einstein radius. For convenience the magnitudes within the Einstein radius are listed in a separate column. We tested that simple aperture photometry on the galaxy, with the ring masked, gives a redshift and *M*/*L* ratio that are within the uncertainties of the fiducial values.

Photometry of the ring is performed by simply summing the flux in apertures. Two apertures are used: one covering both of the red concentrations within the ring and one covering the rest of the ring. No attempt is made to correct for the PSF variation between bands, but the apertures are purposefully made large enough to mitigate these effects. We use the photometry of the ring to derive an approximate redshift, and we caution against using it to determine detailed stellar population parameters of the lensed galaxy. The magnitudes for the two apertures are listed in Supplementary Table 2.

### Prospector fits

The redshift of the lens and its stellar population parameters are determined jointly using the Prospector inference framework^10^, specifically the Prospector-*α* model^54^ and the MIST stellar isochrones^55,56^ from Flexible Stellar Population Synthesis^57^. Prospector-*α* describes the star formation history (SFH) non-parametrically via mass formed in seven logarithmically spaced time bins, and assumes a continuity prior to ensure smooth transitions between bins^58^. We additionally adopt a dynamic SFH(*M*, *z*) prior^59^ that follows the observed cosmic star-formation-rate density, favouring rising SFHs in the early universe and falling SFHs in the late universe, with a mass-based adjustment to reflect downsizing. The model consists of 18 free parameters, including the form of the attenuation curve, and sampling is performed using the dynamic nested sampler dynesty^60^. The parameters for the lens are determined from the photometry inside the ring. We report the posterior median of the inferred physical parameters in Supplementary Table 3, assuming a Chabrier IMF. The uncertainties reflect the 16th and 84th percentiles.

The uncertainties in the redshift and mass may seem suspiciously small given that we only have five photometric datapoints. The reason why the key parameters are so well constrained is that the photometry tells us only one thing, but it does so precisely: there is a large break in the SED at 1.2 μm. The constraints on the redshift and *M*/*L* ratio follow directly from this. We performed two robustness tests to determine how sensitive the results are to the specifics of our methodology. First, removing the SFH prior leads to negligible differences to the redshift and mass, and all the posterior medians are consistent within 1*σ*. The only notable change is that the prior decreases the uncertainty on the star formation rate. This behaviour is expected: at this redshift and mass the prior prefers a falling SFH, consistent with the observed high mass (that is, high previous star formation rate) and low current star formation rate. Second, determining the redshift with the EAZY code^61^ (which uses a pre-rendered set of templates) gives ${z}_{{{{\rm{phot}}}}}=1.9{1}_{-0.17}^{+0.18}$ and no viable secondary solutions, in good agreement with our fiducial value.

The lensed galaxy is modelled in the same way as the lens, except that the scale-dependent SFH prior is not included owing to the lensing magnification. The main goal is to determine the redshift of the lensed galaxy. For completeness we list stellar population parameters for the two apertures on the ring as well in Supplementary Table 3, although they are not used in the analysis.

### Obscured star formation

The low star formation rate of JWST-ER1g derived above implies a low gas surface density, and hence a low contribution of gas to the total mass budget within the Einstein ring. However, the Prospector fits do not provide strong constraints on the amount of star formation that is optically thick. The field has been observed with Spitzer/MIPS, as part of the S-COSMOS survey^62^, and we use the 24 μm data to assess whether JWST-ER1g has a hidden obscured star burst.

The S-COSMOS 24 μm image is shown in Supplementary Fig. 3. The galaxy is not detected. We determine an upper limit to the star formation rate from a redshift-dependent relation between observed 24 μm flux and total infrared luminosity that was calibrated with Herschel data^63,64^. The 3*σ* upper limit is 63 *M*_⊙_ yr^−1^.

### Comparison to other *z* ≈ 2 galaxies

As noted in the main text, JWST-ER1g is a typical example of the class of massive, quiescent *z* ≈ 2 galaxies. This is demonstrated explicitly in Supplementary Fig. 4. Supplementary Fig. 4a shows that the galaxy falls in the quiescent region of the UVJ diagram. The boundaries are the averages of the *z* = 1.75 and *z* = 2.25 limits determined for the NEWFIRM Medium Band Survey^9^. It is relatively red within the quiescent region, indicating an old age and/or some dust, as also implied by the Prospector fit. In Supplementary Fig. 4b the galaxy’s size is compared to the canonical size–mass relations^16^ for quiescent and star-forming galaxies, again taking the average of the listed relations for *z* = 1.75 and *z* = 2.25. The galaxy falls on the relation for quiescent galaxies.

### Lensing mass

The mass within the Einstein radius is given by:

1 $$M( < \theta )=\frac{{\theta }^{2}{c}^{2}{D}_{{{{\rm{l}}}}}{D}_{{{{\rm{s}}}}}}{4G{D}_{{{{\rm{ls}}}}}},$$

with *θ* the observed Einstein radius in radians, *c* the speed of light, *D*_l_ the angular diameter distance to the lens, *D*_s_ the angular diameter distance to the source and *G* the gravitational constant. The parameter *D*_ls_ is the distance between the lens and the source, which is:

2 $${D}_{{{{\rm{ls}}}}}={D}_{{{{\rm{s}}}}}-\frac{1+{z}_{{{{\rm{l}}}}}}{1+{z}_{{{{\rm{s}}}}}}{D}_{{{{\rm{l}}}}},$$

with *z*_l_ the redshift of the lens and *z*_s_ the redshift of the source in a flat Universe^65^. The uncertainties are determined numerically, by drawing values of *z*_s_, *z*_l_ and *θ* from their probability distributions and calculating *M*(<*θ*) for each set of draws.

The high lens mass is driven by the large diameter of the Einstein ring combined with the relatively high redshift of the lens. Forcing *z*_l_ = 1.5 (which is outside of the full posterior distribution of 5,000 samples) lowers the mass to *M*_lens_ = 4.1 × 10^11^ *M*_⊙_, but also lowers the derived Chabrier stellar mass to *M*_stars_ = 0.6 × 10^11^ *M*_⊙_. The ratio of the lensing mass to the Chabrier mass is ~7, which is very similar to the results for *z* = 1.94.

The source redshift is the most uncertain parameter in equation (1). The lensing mass is lower for higher source redshifts, but is 3.7 × 10^11^ *M*_⊙_ even for *z*_s_ = 5. The uncertainty in the source redshift also causes an asymmetry in the error distribution of *M*_lens_, with a tail to very high masses. This is because the mass increases rapidly when *z*_s_ ≈ *z*_l_: the mass is >10^12^ *M*_⊙_ if *z*_s_ < 2.5, and reaches 4 × 10^12^ *M*_⊙_ for *z*_s_ = 2.1.

### Dark matter contribution

The projected dark matter mass within the ring can be calculated by integrating a NFW profile^21^ along a cylinder with a radius of 6.6 kpc (ref. ^66^). The scaling $\log c=0.81-0.09(\log {M}_{{{{\rm{vir}}}}}-12)$ is used to determine the concentration as a function of halo mass, with *M*_vir_ the virial mass^67^. The resulting relation between projected dark matter mass within the ring and total halo mass is shown in Supplementary Fig. 5.

The relation is shallow, owing to the decreasing concentration with halo mass. We estimate the dark matter contribution to the lensing mass from the halo mass–stellar mass relation^22^. We find ${M}_{200}=1.{0}_{-0.5}^{+2.6}\times 1{0}^{13}$ *M*_⊙_, where *M*_200_ is the mass within the radius where the overdensity is a factor of >200, with the relatively large uncertainty driven by the steepness of the relation in this regime. The corresponding projected dark matter mass within 6.6 kpc is ${M}_{{{{\rm{dm}}}}}=2.{6}_{-0.7}^{+1.6}\times 1{0}^{11}$ *M*_⊙_ for a NFW halo.

The solid horizontal line indicates the difference between the lensing mass and the stellar mass of JWST-ER1g for a Chabrier IMF. To explain the missing mass entirely with dark matter, the NFW halo mass would have to be ~7 × 10^13^ *M*_⊙_. Halos of this mass at *z* = 2 are progenitors of clusters at *z* = 0. The number density of halos with *M*_200_ > 7 × 10^13^ *M*_⊙_ at *z* = 1.94 is 2 × 10^−7^ h^−3^ Mpc^−3^, corresponding to 1.4 in the redshift range 1.75 < *z* < 2.25 in the 0.35 deg^2^ of the available COSMOS-Web area^68^. Halos with slightly lower masses are of course more common, and still consistent with the lensing constraints. The lower 1*σ* bound on the lensing mass corresponds to a halo mass of *M*_200_ > 3 × 10^13^ *M*_⊙_ (Supplementary Fig. 5), and there are ~15 such halos in the COSMOS-Web area.

### Environment of JWST-ER1

Gravitational lensing is sensitive to the weighted integral of all mass between the source and the observer, and we briefly consider whether nearby galaxies or structures along the line of sight could contribute to the mass. We also consider whether JWST-ER1g is the central galaxy of the progenitor of a cluster (see above). The immediate environment of JWST-ER1 is shown in Supplementary Fig. 6, as generated from the NIRCam F115W, F277W and F444W images. The region does not stand out in any way; the galaxy is either isolated or in a sparse group, but not in a massive cluster. Furthermore, there are no other bright galaxies projected along the line of sight. We infer that the contributions from other galaxies to the 6.7 × 10^11^ *M*_⊙_ mass within the Einstein radius are almost certainly negligible.

### Supplementary information

Supplementary Information Supplementary Tables 1–3 and Figs. 1–6.

## Data Availability

The COSMOS-Web data are publicly available from the STScI MAST Archive.

## Code availability

We have made use of standard data analysis software in the Python and IRAF environments, and the publicly available code GALFIT^53^.
