# Supplementary material for: A massive compact quiescent galaxy at z = 2 with a complete Einstein ring in JWST imaging
Source: Nat Astron. 2023 Oct 19;8(1):119–25. doi: 10.1038/s41550-023-02103-9 (PMC10803264; doi:10.1038/s41550-023-02103-9)
Supplement: Supplementary file 1 — Supplementary Tables 1–3 and Figs. 1–6. [file 41550_2023_2103_MOESM1_ESM.pdf]

nature astronomy

Article https://doi.org/10.1038/s41550-023-02103-9

A massive compact quiescent galaxy at $z = 2$ with a complete Einstein ring in JWST imaging

In the format provided by the authors and unedited

A massive compact quiescent galaxy at z = 2 with a complete Einstein ring in JWST imaging

Pieter van Dokkum[1], Gabriel Brammer[2,3], Bingjie Wang[4], Joel Leja[4,5], Charlie Conroy[6]

[1] *Department of Astronomy, Yale University, New Haven, CT 06511, USA*
[2] *Cosmic Dawn Center (DAWN), Denmark*
[3] *Niels Bohr Institute, University of Copenhagen, Jagtvej 128, DK-2200 Copenhagen N, Denmark*
[4] *Department of Astronomy & Astrophysics, The Pennsylvania State University, University Park, PA 16802, USA*
[5] *Institute for Computational & Data Sciences, The Pennsylvania State University, University Park, PA 16802, USA*
[6] *Harvard-Smithsonian Center for Astrophysics, 60 Garden Street, Cambridge, MA, USA*

Supplementary Information

Filter	Total	In ring
F814W	25.97 ± 0.14	26.22 ± 0.14
F115W	23.36 ± 0.08	23.61 ± 0.08
F150W	22.14 ± 0.05	22.39 ± 0.05
F277W	20.95 ± 0.05	21.20 ± 0.05
F444W	20.43 ± 0.05	20.68 ± 0.05

Supplementary Table 1: Photometry of the lens (AB mag).

Filter	Blue ring	Red knots
F814W	24.55 ± 0.06	27.53 ± 0.35
F115W	23.85 ± 0.09	26.66 ± 0.29
F150W	23.24 ± 0.07	25.40 ± 0.11
F277W	22.31 ± 0.05	23.58 ± 0.05
F444W	21.89 ± 0.05	22.70 ± 0.05

Supplementary Table 2: Photometry of the source (AB mag).

	Lens	Blue ring	Red knots
z	$1.94^{+0.13}_{-0.17}$	$2.89^{+0.27}_{-0.98}$	$2.98^{+0.42}_{-0.47}$
log M/M$_\odot$	$11.03^{+0.09}_{-0.13}$	$10.43^{+0.22}_{-0.56}$	$10.63^{+0.16}_{-0.18}$
SFR [M$_\odot$ yr^{-1}]	4^{+19}_{-3}	64^{+36}_{-25}	19^{+48}_{-16}
age [Gyr]	$1.9^{+0.3}_{-0.6}$	$0.8^{+0.3}_{-0.5}$	$1.1^{+0.4}_{-0.4}$

Supplementary Table 3: Inferred parameters.

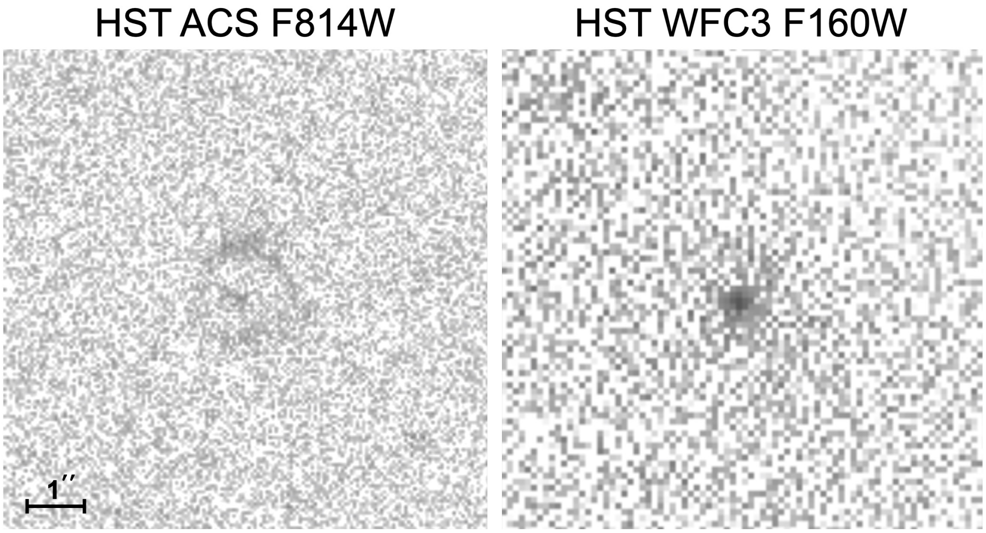

Supplementary Figure 1: **HST data of JWST-ER1.** Pre-JWST high resolution imaging shows the ring in ACS F814W and the galaxy in WFC3 F160W. The system could have been flagged as a candidate Einstein ring.

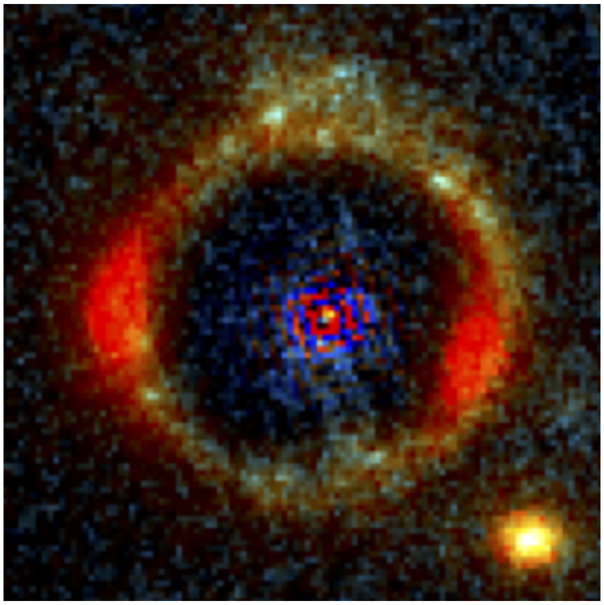

Supplementary Figure 2: **Symmetries in the ring.** This color image was created from the F150W and F444W images, after subtracting the `galfit` model of the central galaxy. The image spans $2.5'' \times 2.5''$. There are several candidate multiply-imaged features along the ring. The two red knots are very bright in F444W and are stretched into mirrored arcs. This would be difficult to explain by any other mechanism than gravitational lensing.

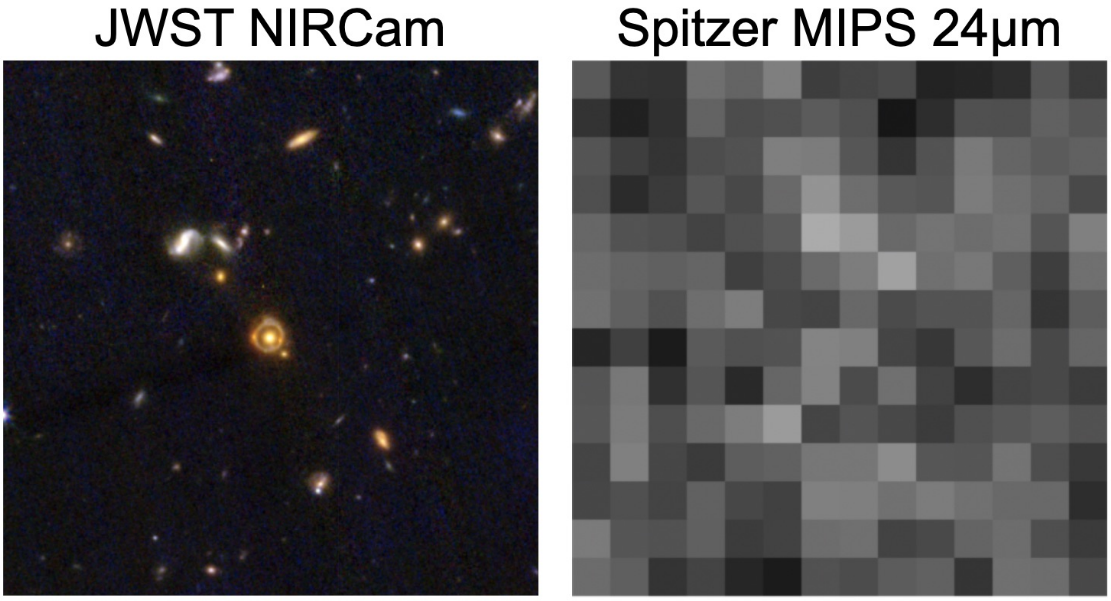

Supplementary Figure 3: **Non-detection at 24 micron.** The $30'' \times 30''$ region around JWST-ER1 as observed with Spitzer/MIPS at 24 μm. The galaxy is not detected, and the upper limit on the star formation rate of the lensing galaxy is 63 $M_\odot$ yr^{-1}.

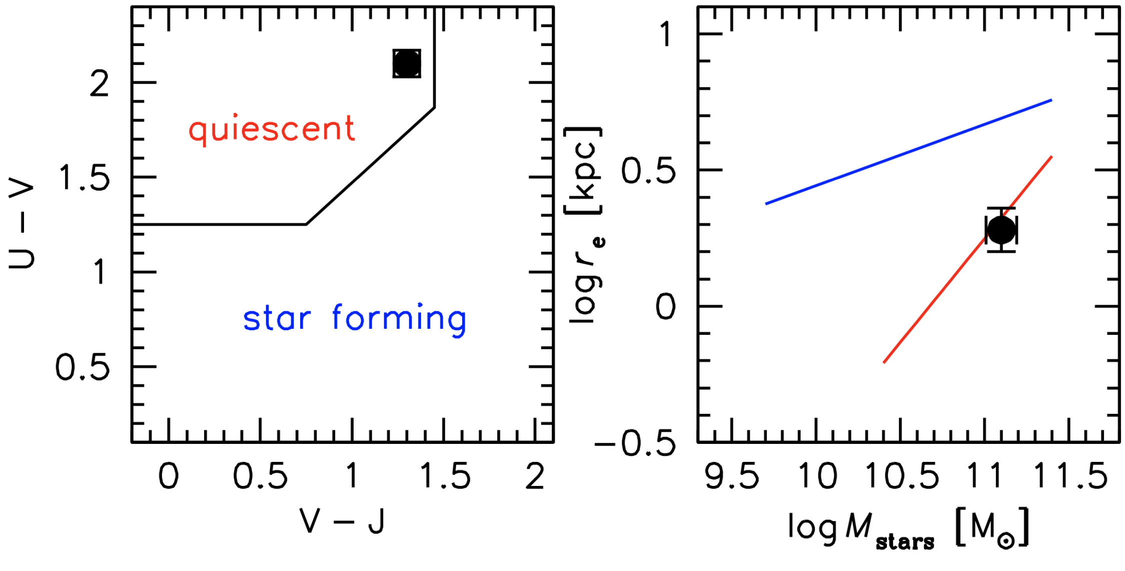

Supplementary Figure 4: **JWST-ER1g in context.** a) Location in the UVJ diagnostic diagram for $z \sim 2$. The galaxy falls in the quiescent region. b) Location in the size-mass diagram for $z \sim 2$. It falls on the size-mass relation for quiescent galaxies. Data points are measurements $\pm$ sd.

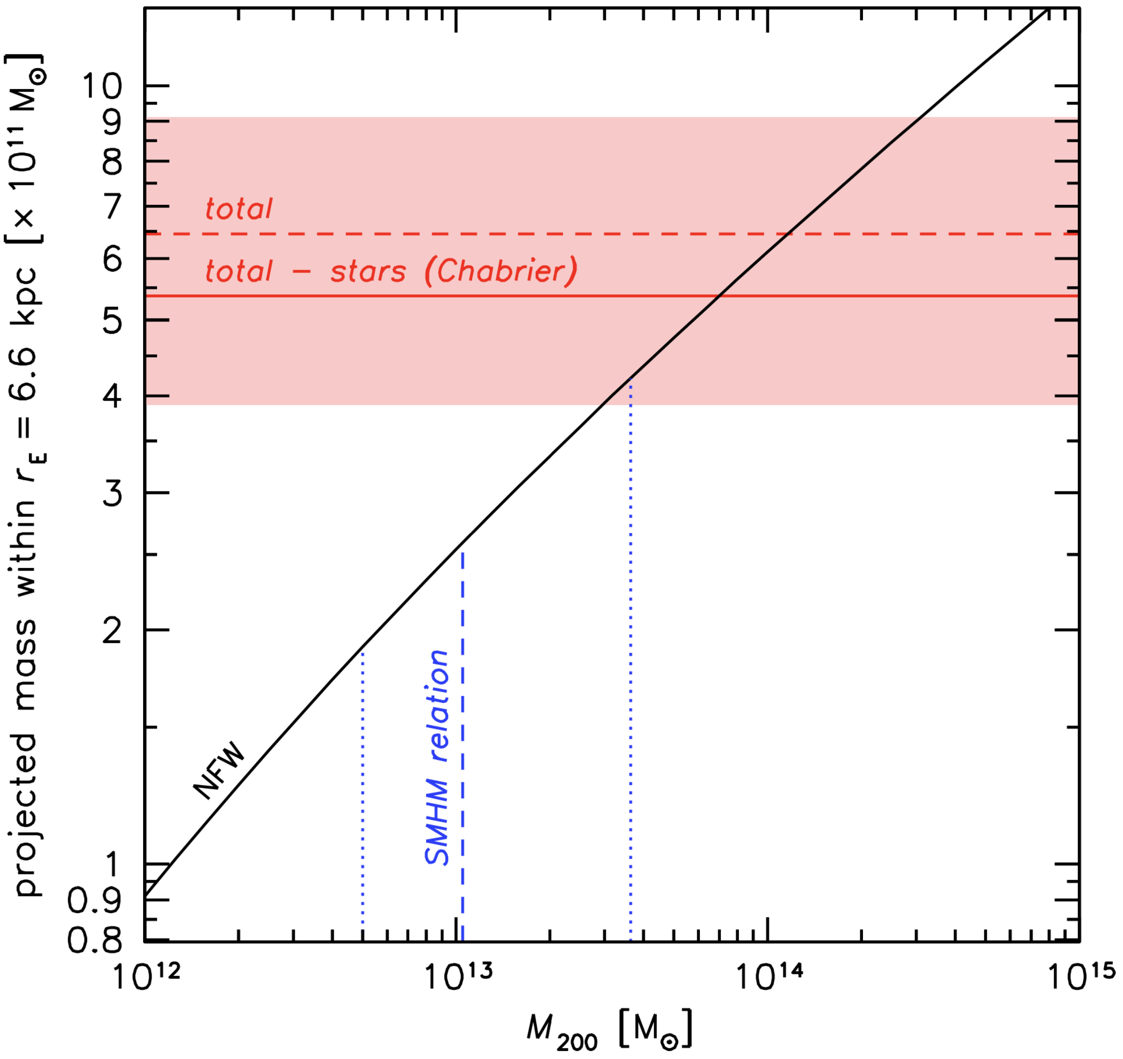

Supplementary Figure 5: **Relation between projected mass within the Einstein radius and total halo mass.** The dashed horizontal line indicates the total lensing mass. The solid horizontal line is the remaining mass after subtracting the stellar mass, for a Chabrier IMF and with the band indicating the $\pm1\sigma$ uncertainty. The blue vertical lines show the expected halo mass from the $z = 2$ stellar mass – halo mass (SMHM) relation and its uncertainty. The solid black line is the expected relation for NFW halos at $z = 1.94$. The galaxy has more mass within the Einstein radius than expected from a Chabrier IMF, the SMHM relation, and an NFW profile.

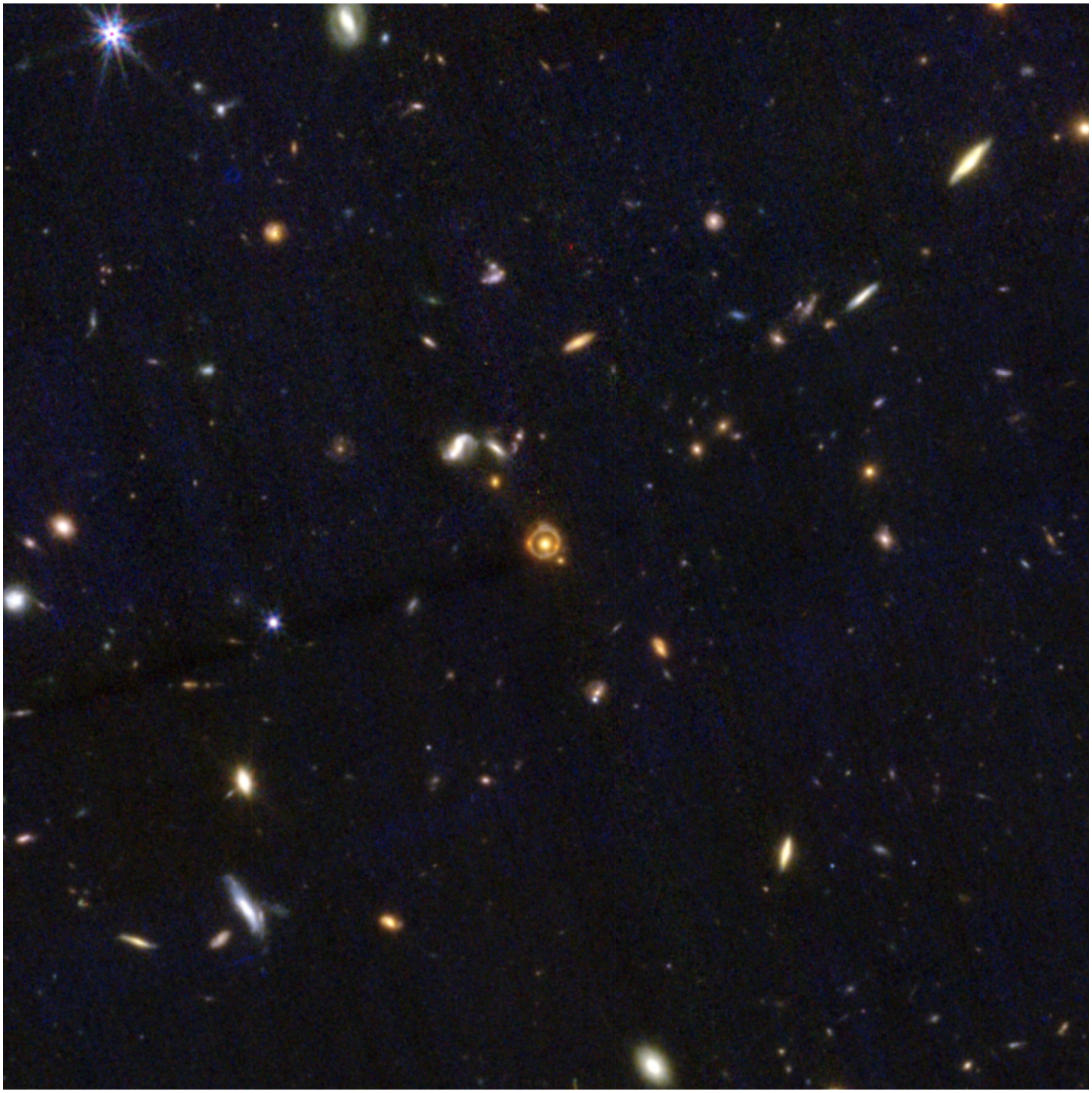

Supplementary Figure 6: **Environment of the lens.** The $1' \times 1'$ region of the COSMOS-Web mosaic centered on JWST-ER1. The object appears to be relatively isolated; it may of course be part of a group, but it is not near the central regions of a rich cluster.
